# Predicting the Recurrence of Differentiated Thyroid Cancer Using Whale Optimization-Based XGBoost Algorithm

**DOI:** 10.3390/diagnostics15131684

**Published:** 2025-07-02

**Authors:** Keshika Shrestha, H. M. Jabed Omur Rifat, Uzzal Biswas, Jun-Jiat Tiang, Abdullah-Al Nahid

**Affiliations:** 1 Electronics and Communication Engineering Discipline, Khulna University, Khulna 9208, Bangladesh; keshika2002@gmail.com (K.S.); jabedomur.rifat@gmail.com (H.M.J.O.R.); 2 Centre for Wireless Technology, CoE for Intelligent Network, Faculty of Artificial Intelligence & Engineering, Multimedia University, Persiaran Multimedia, Cyberjaya 63100, Selangor, Malaysia

**Keywords:** thyroid cancer, recurrence prediction, XGBoost, WHALE Optimization Algorithm (WOA), hyperparameter optimization, feature selection

## Abstract

**Background/Objectives:** Differentiated Thyroid Cancer (DTC), comprising papillary and follicular carcinomas, is the most common type of thyroid cancer. This is highly infectious and increasing at a higher rate. Some patients experience recurrence even after undergoing successful treatment. Early signs of recurrence can be hard to identify, and the existing health care system cannot always identify it on time. Therefore, predicting its recurrence accurately and in its early stage is a significant clinical challenge. Numerous advanced technologies, such as machine learning, are being used to overcome this clinical challenge. Thus, this study presents a novel approach for predicting the recurrence of DTC. The key objective is to improve the prediction accuracy through hyperparameter optimization. **Methods:** In order to achieve this, we have used a metaheuristic algorithm, the whale optimization algorithm (WOA) and its modified version. The modifications that we introduced in the original WOA algorithm are a piecewise linear chaotic map for population initialization and inertia weight. Both of our algorithms optimize the hyperparameters of the Extreme Gradient Boosting (XGBoost) model to increase the overall performance. The proposed algorithms were applied to the dataset collected from the University of California, Irvine (UCI), Machine Learning Repository to predict the chances of recurrence for DTC. This dataset consists of 383 samples with a total of 16 features. Each feature captures the critical medical and demographic information. **Results:** The model has shown an accuracy of 99% when optimized with WOA and 97% accuracy when optimized with the modified WOA. **Conclusions:** Furthermore, we have compared our work with other innovative works and validated the performance of our model for the prediction of DTC recurrence.

## 1. Introduction

Thyroid cancer is one of the most prevalent endocrine malignancies, with differentiated thyroid cancer (DTC) accounting for approximately 90% of all cases [1]. DTC, which includes papillary and follicular thyroid carcinomas, is rising in incidence and is known for its relatively favorable prognosis due to advancements in treatment, including surgery, radioactive iodine therapy, and thyroid hormone suppression therapy [2,3]. Despite these improvements, recurrence remains a major clinical issue, adversely affecting both prognosis and quality of life. Haugen et al. [4] reported that nearly 75% of high-risk DTC patients experienced recurrence, while a study by Hollenbeak et al. found a recurrence rate of 39% among 2883 elderly patients [5]. Zahedi et al. [6] further identified key risk factors such as age, cancer stage, and treatment history that significantly influence recurrence, with gender also playing a role, with women showing a recurrence rate of 2.2%, while men have a rate of 8.5%. Consequently, early identification and accurate prediction of recurrence are critical for improving patient outcomes and guiding treatment strategies.

The primary objective of this study is to develop a novel framework that combines the whale optimization algorithm (WOA) with the Extreme Gradient Boosting (XGBoost) model to accurately predict DTC recurrence. The proposed model aims to enhance predictive performance through optimization using both feature selection and hyperparameter tuning. This ultimately supports improved clinical decision making and long-term patient management.

Predicting recurrence in DTC is complex due to the involvement of various clinical and pathological factors. Traditional methods, including medical history, physical examinations, and pathological assessments, often fall short in capturing these intricate relationships [7]. As a result, advanced predictive tools are needed to overcome these limitations and enhance decision-making. Recently, machine learning (ML) techniques have emerged as promising tools in this domain. ML algorithms can analyze large, complex datasets and detect patterns that are not easily discernible through conventional methods. For instance, Borozooei et al. [8] employed classic ML models such as K-nearest neighbors (KNN), support vector machines (SVMs), tree-based models, and artificial neural networks (ANNs) to predict recurrence in DTC patients with an average accuracy of 93%. Chattopadhya [9] employed multiple linear regression (MLR) and reported an accuracy of 78.32%. In contrast, Setiawan [10] utilized a random forest (RF) model, achieving an accuracy of 96%, while Clark et al. [11] also applied the RF algorithm, obtaining an accuracy of 95%. Similarly, other ML techniques, especially the XGBoost model, have shown incredible performance in medical prediction tasks by not only improving the robustness but also by improving accuracy [12]. Latif et al. [13] developed a hybrid model that integrated ensemble stacking with bidirectional feature selection for the accurate diagnosis of thyroid disorders. It primarily focused on initial detection or diagnosis and not recurrence prediction which is a distinct and more critical challenge. It also lacked the specialized design needed for recurrence prediction in a cancer-specific context. Similarly, another study conducted by Oture et al. [14] also focuses on improving the diagnosis of thyroid disease using advanced ML methodologies to provide a more reliable and efficient diagnostic accuracy. This model also does not address the prediction of DTC recurrence which is crucial for long-term patient management. Additionally, these ML models also did not incorporate features specifically associated with recurrence risk. There are also some studies, such as Afshan et al. [15] and Akhtar et al. [16], that have reported higher accuracy scores using ensemble methods and oversampling techniques like ADASYN. However, they failed to address model simplicity, clinical interpretability, or the incorporation of optimization techniques.

Optimization is an important component of building high-performing ML models. Optimization algorithms adjust model parameters to improve accuracy and reliability. Among various approaches, the WOA has gained attention for mimicking the hunting behavior of humpback whales. This algorithm has also shown strong performance in solving diverse mathematical and structural optimization problems [17]. However, its direct application in medical data analysis specifically for predicting DTC recurrence might show limited success due to issues like premature convergence and getting trapped in local optima. These limitations suggest the need for modifications to WOA to make it more effective in this context.

To address these challenges, this study introduces a novel predictive framework that integrates a modified whale optimization algorithm (MWOA) with the XGBoost model. The proposed MWOA improves the balance between exploration and exploitation by enhancing the search mechanism of the WOA using strategies such as weight inertia and Piecewise Linear Chaotic Weight (PWLCW). This modification is expected to optimize feature selection and hyperparameter tuning, thereby increasing the accuracy and robustness of recurrence prediction in DTC patients.

## 2. Materials and Methods

**Figure 1:**
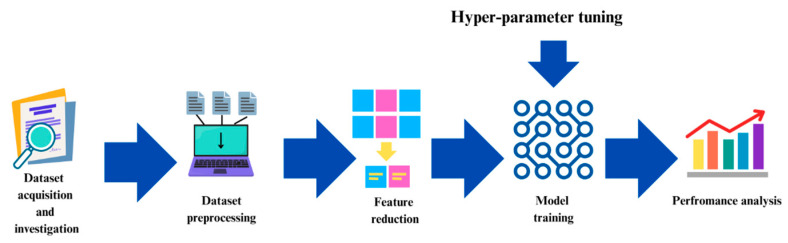
Overall methodology of this study.

For our work, we have preprocessed the DTC dataset collected from the UCI Machine Learning Repository [8] and also carried out feature reduction on the same dataset. Then we have optimized the XGBoost model using WOA and its modified version. An overview of our methodology is illustrated in Figure 1, which we have divided into five parts named data acquisition, data preprocessing, and feature reduction using the modified WOA, model training, hyper-parameter tuning, and performance analysis.

### 2.1. Dataset Acquisition

The dataset for this research has been collected from UCI Machine Learning Repository [8]. The primary purpose of this dataset is to predict the chances of recurrence for DTC. A total of 383 samples are present in this dataset with a total of 16 features, capturing critical medical and demographic information. All of these attributes are strings. An overview of the dataset is presented in Table 1.

The dataset was collected over a span of 15 years, with each patient being followed for a minimum of 10 years. Among the 383 patients, 312 were female and 71 were male, with a higher recurrence rate observed in females. This dataset primarily consists of early-stage thyroid cancer patients where distant metastasis is extremely uncommon, occurring in only 4.7% of cases. Recurrence is observed in approximately 31.3% of patients. This indicates a significant proportion of cases where the cancer returns after treatment. Around 208 patients had an excellent response to treatment, with 114 patients showing incomplete responses and 61 patients showing an indeterminate response. This dataset was gathered as part of research in the field of AI and medicine and contains no missing values. Leveraging this dataset, we aim for an improvement in the early detection of recurrence and optimization of patient monitoring, ultimately contributing to better management and prognosis of DTC.

### 2.2. Dataset Preprocessing

A very important step in data analysis is the data preprocessing, which includes cleaning, organizing, encoding, and decoding of the datasets [18]. Our initial step for the preprocessing part was inspection of the dataset. The dataset was checked for any missing or duplicated data and none of the columns had it. All the 16 features had categorical values. Hence, we have performed a categorical conversion for those 16 features. These categorical variables were encoded using hot encoding, which transforms categorical data into a binary matrix representation. Each category in the original feature is converted into a new binary feature, with a value of 1 indicating the presence of the category and 0 indicating its absence.

### 2.3. Feature Selection

In our research we have used basic-WOA and modified-WOA algorithms to perform feature selection. WOA is a metaheuristic algorithm, which has gained prominence in feature selection [19]. These algorithms efficiently navigate vast search spaces, balancing exploration and exploitation to identify optimal solutions. Their ability to deliver reliable results with reduced computational overhead makes them increasingly valuable in feature selection applications [20].

#### 2.3.1. Basic Whale Optimization Algorithm

**Figure 2:**
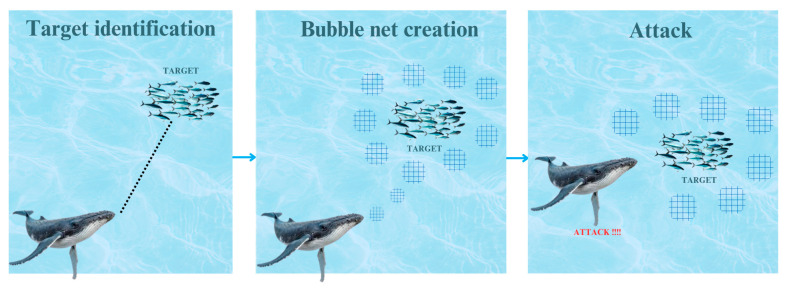
Bubble-net hunting strategy.

The whale optimization algorithm is a nature-inspired meta-heuristic algorithm, which mimics the hunting behavior of the humpback whale [17]. The unique hunting strategy of the humpback whale is depicted in Figure 2. Its mathematical model has three aspects: search, encircling prey, and attack.

(a) Encircling prey: The humpback whale can locate its prey and moves towards it. This is known as encircling prey. The best position is the position of the whale close to the prey that leverages the movement of the other whales. This behavior is represented as follows:

(1) $$\vec{D}=\left|\vec{C}\cdot {\vec{X}}^{*}(t)-\vec{X}(t)\right|,$$

(2) $$\vec{X}\left(t+1\right)=\vec{X^{*}}\left(t\right)-\vec{A}\cdot \vec{D},$$

(3) $$\vec{A}=2\vec{a}\cdot \vec{r}-\vec{a},$$

(4) $$\vec{C}=2\cdot \vec{r}$$

where

$\vec{X^{*}}$ is the best position;
$\vec{X}$ is the current position;
$\vec{A}$ and $\vec{C}$ are coefficient vectors;
$\vec{a}$ is linearly decreased from 2 to 0 over the course of iterations;
$\vec{r}$ is a random vector;
$\vec{D}$ is the distance between the best and current positions.

(b) Attacking prey: This refers to the exploration behavior presented by the humpback whale. The whale moves closer to the best solution found so far. The humpback whale has unique path movement, i.e., the bubble-net hunting strategy. This hunting strategy is implemented in two ways: the shrinking encircling mechanism and spiral position updating, and its mathematical model is represented in the equations below:

(5) $$\vec{X}\left(t+1\right)=\vec{D^{\prime }}\cdot e^{bl}\cdot \cos\left(2\pi l\right)+\vec{X^{*}}(t),$$

(6) $$\vec{D^{\prime }}=\left|{\vec{X}}^{*}(t)-\vec{X}(t)\right|,$$

(7) $$\vec{X}(t+1)=\left\{\begin{array}{c} \vec{X^{*}}(t)-\vec{A}\cdot \vec{D}\;\;\;\;\;\;\;\;\;\;\;\;\;\;\;\;\;\;\;\;\;\;\;\;\;\;\;\;\;\;if\;p<0.5\\ \vec{D’}\;\cdot e^{bl}\cdot cos(2\pi l)+\vec{X^{*}}(t)\;\;\;\;\;\;\;\;\;\;\;if\;p\geq \;0.5 \end{array}\right.$$

where

$\vec{D^{\prime }}$ is the distance of ith whale to the best solution obtained so far;
$\vec{X}\left(t+1\right)$ indicates the updated position of whales during optimization;
*b* defines the logarithmic spiral shape;
*l* is a random number;
*p* determines whether the population encircles or spirals to attack.

(c) Searching prey: To improve the global search ability of the algorithm, the whale population surrounds the prey when |$\vec{A}$| ≤ 1 or a search agent is updated randomly, and its mathematical model is as follows:

(8) $$\vec{D}=\left|\vec{C}{\cdot \vec{X}}_{rand}-\vec{X}\right|,$$

(9) $$\vec{X}\left(t+1\right)=\vec{X_{rand}}-\vec{X}\cdot \vec{D}$$

where $\vec{X_{rand}}$ is the random position vector chosen from the current population.

Although the WOA is a powerful metaheuristic algorithm, it has certain limitations. The WOA sometimes settles for a good enough solution instead of finding the absolute best one. This happens when it locks onto a suboptimal answer too soon. Another limitation of the WOA is that it is sensitive to parameter selection, and it does not always balance the exploration and exploitation phases [21].

#### 2.3.2. Modified Whale Optimization Algorithm

In order to overcome the limitations of the basic WOA, we have proposed some modifications to the original WOA. The modifications that we have proposed are as follows:

(a) Inertia weight (*w*)

Inertia weight is a crucial parameter often incorporated into optimization algorithms to balance the exploration (global search) and exploitation (local search) phase. This was originally introduced in particle swarm optimization (PSO) to enhance convergence and prevent premature stagnation [22]. The equation for inertia weight is represented in the equation below, and by integrating it into WOA, the algorithm gains improved adaptability in the search process.

(10) $$w=\left|\cos\left(\frac{nt\pi }{t_{max}}\right)\right|,$$

where

*w* is the inertia weight coefficient;
*t* is the current iteration number in the optimization algorithm;
$t_{max}$ is the maximum number of iterations in the algorithm;
*n* is a constant parameter that affects the frequency of oscillation.

This approach improves exploration because in the early iterations a higher inertia weight allows the search agents (whales) to cover a larger area of the solution space. This helps them avoid getting stuck too soon and increases the chances of finding the best possible solution. As the iterations progress, reducing the inertia weight helps refine the search around optimal solutions, ensuring convergence. This enhances the exploitation phase. The standard WOA can sometimes get trapped in local optima. The inertia weight mechanism helps mitigate this by maintaining diversity among search agents.

The new update mechanism of the modified WOA can be written as follows:

(11) $$X_{i}\left(t+1\right)=w\cdot X_{i}\left(t\right)+\vec{C}\cdot {(X^{*}-X}_{i}(t))\;,$$

where

$X_{i}\left(t\right)$ represents the position of the whale at iteration t;
$X^{*}$ is the best solution so far;
$\vec{C}$ is a coefficient vector controlling the balance between exploration and exploitation;
*w* is the inertia weight.

Several studies have demonstrated that incorporating inertia weight improves the WOA’s performance. Guo et al. [23] integrated w into WOA for feature selection, and an enhanced classification accuracy with reduced feature subsets was mentioned. Similarly, Chao et al. [24] found that an inertia-weighted WOA outperformed the standard WOA in solving complex benchmark functions and engineering optimization problems. Therefore, adding w to WOA not only refines its search mechanism but also improved the accuracy. It gives a better convergence rate and more reliable classification outcomes.

(b) Piecewise linear chaotic map

Piecewise linear chaotic maps (PWLCMs) are mathematical functions characterized by linear segments, designed to exhibit chaotic behavior. Due to their simplicity and robust chaotic properties, PWLCMs are widely utilized in various fields, including cryptography, optimization algorithms, and image processing [25]. The equation below mathematically represents PWLCMs:

(12) $$XY_{i}=F(XY_{i}-\beta )\left\{\begin{array}{c} XY_{i-1}/\;\beta ,\;\;0<XY_{i}<\beta \\ (XY_{i-1}-\beta )/(0.5-\beta ),\;\;\beta \leq XY_{i-1}<0.5\\ F(1-XY_{i-1},\beta ),\;\;0.5\;\leq XY_{i-1}<1 \end{array}\right.,$$

where

$XY_{i}$ and *β* refers to the initial condition and control parameters, respectively.

The position of the search agent ($XY_{i}$) is updated based on the position of the previous agent ($XY_{i-1}$) and a parameter *β*. The update occurs depending on the value of $XY_{i-1}$. In the context of optimization algorithms, integrating PWLCMs can enhance performance by improving population diversity and preventing premature convergence. For instance, the Beluga Whale Optimization Algorithm (BWOA), which was inspired by the social hunting behavior of beluga whales, has been improved by incorporating PWLCMs. This integration refines the algorithm’s ability to explore and exploit the search space effectively, leading to more accurate and reliable solutions [26].

In summary, the integration of PWLCMs into optimization algorithms like the WOA introduces beneficial chaotic dynamics. This integration enhances the algorithm’s exploration and exploitation capabilities, leading to improved performance across various applications, including complex optimization problems and real-time monitoring systems [21].

### 2.4. Model Trained Using XGBoost Classifier Algorithm

XGBoost 2.1.4 is an advanced implementation of the gradient boosting framework designed for efficiency, scalability, and improved predictive performance. It is based on the principle of boosting, where weak learners (decision trees) are sequentially improved by minimizing a differentiable loss function using gradient descent optimization [27]. Unlike traditional boosting methods, XGBoost introduces a second-order Taylor expansion of the loss function, which allows for precise gradient and Hessian (second-order derivative) calculations, resulting in more accurate weight updates [28]. This optimization technique enhances convergence speed and reduces computational cost compared to conventional gradient boosting methods. The efficiency of XGBoost has been demonstrated in numerous applications, including medical diagnosis, fraud detection, and financial risk prediction [12]. Studies have shown that XGBoost outperforms traditional classifiers such as RF, SVM, and ANN in structured data problems due to its superior feature selection, interpretability, and computational efficiency [29].

#### Hyperparameter Tuning

Hyperparameters are used in machine learning to control the action, complexity, and even the performances of the models. When tuning such hyperparameters in gradient boosting frameworks such as XGBoost, the performance of the model and its generalization ability and computational proficiency are affected [28]. Here, most critical are ‘max_depth’, ‘learning_rate’, and ‘n_estimators’, which are features influencing the model in different ways.

There were also two hyperparameters used in this model, max_depth and the number of trees, with max_depth controlling the depth of the trees and therefore the complexity of the model. The greater depth also allows the model to better capture interactive features and relationships within the data set. However, this added complexity comes at a price. The basic problem of overfitting occurs when the learning algorithm begins to capture noise and outliers in the training data instead of learning the underlying patterns that generalize well to unseen data. This results in a model that performs exceptionally well on the training set but poorly on new, unseen data, compromising its ability to generalize effectively. On the other hand, a smaller tree depth limits the model’s capability of learning complicated patterns thus leading to under fitting. In such cases, the capacity of the model to apply particular formats of nature may be distorted, which leads to the general loss of reliability. Except for the fact that one should be cautious to not go too deep in the trees, an appropriate level of tree depth must be reached to have high model accuracy while maintaining an acceptable level of generalization [28,30].

The learning rate in XGBoost acts like a “step size” for updates. It determines how much each tree contributes to the final prediction. When you lower the learning rate, the model makes smaller, more careful updates. This helps it generalize better and become more robust. However, this benefit comes with a trade-off which is that you will need more trees (and more iterations) to achieve good performance. This further increases the computational cost and training time. In short, a smaller learning rate makes the model more reliable, but it also requires more resources to get there. On the other hand, a higher learning rate increases the rate of learning by giving each tree component a stronger mechanism towards the prediction. Although the use of such an approach saves time during training the model, the negative aspect of it is that the model may converge to a suboptimum solution in favor of the global optimum. The proper adjustment of this parameter plays a big role in determining the ideal combination between computational time and prediction accuracy [19,27].

The parameter n_estimators determine the general capacity of the constructed ensemble, fixing the number of trees that compose the structure. An increase in the number of trees normally enhances the accuracy of the model in order to achieve the capability of capturing more detailed patterns in the dataset. However, the problem that results from having too many trees is overfitting particularly when compounded with high tree depth or a large learning rate. When using more trees in the model, it increases complexity and leads to higher computational demands. On the other hand, using too few trees can risk underfitting, as the ensemble may not capture enough detail in the data to make accurate predictions. Striking the right balance is key. Therefore, there should be enough trees to learn the patterns effectively, but not so many that the model becomes overly complex or computationally expensive. To maintain the best results, this parameter must be balanced in order to have optimum performance and optimum efficiency [20]. We have used a modified WOA as well as the original WOA for hyperparameter tuning. The optimized XGBoost hyperparameters by the modified WOA with their values are as follows: max_depth 6, learning_rate 0.0128, and n_estimators 135. Similarly, the optimized XGBoost hyperparameters by the basic WOA with their values are as follows: max_depth 3.975, learning_rate 0.292, and n_estimators 39.008. This hyper tuning of the parameters of the XGBoost model helps regulate its complexity and enhances regularization.

### 2.5. Performance Analysis

For the evaluation of the performance of our model, several performances metrics have been utilized including accuracy, precision, recall, and the F1 score. Accuracy measures how many correct predictions the model achieved compared to the total number of predictions. It is a simple way to see how often the model is correct. Precision represents the proportion of true positives to all predicted positive instances. Recall calculates the proportion of correctly identified positive cases to the actual number of positive instances. The F1 score combines precision and recall into a single performance metric, calculated as their harmonic mean. It is particularly useful when precision and recall are of equal importance, providing a balanced measure of the model performance.

## 3. Results

**Figure 3:**
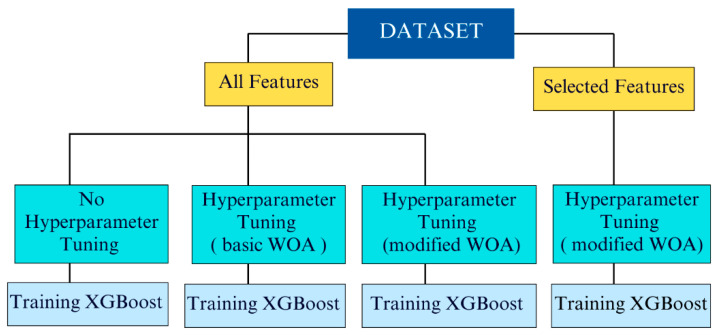
Visualization of model training process.

In this section the outcomes of this research have been presented sequentially. An overview of our training process is illustrated in Figure 3.

We conducted our analysis using all available features as well as using only a selected subset of features. A summary of the results is shown in Table 2.

### 3.1. Performance of XGBoost Model with All Features

**Figure 4:**
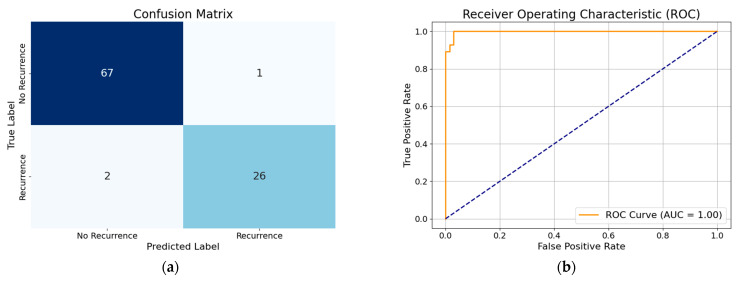
(**a**) Confusion matrix of XGBoost model with all features; (**b**) ROC curve of XGBoost model with all features.

**Figure 5:**
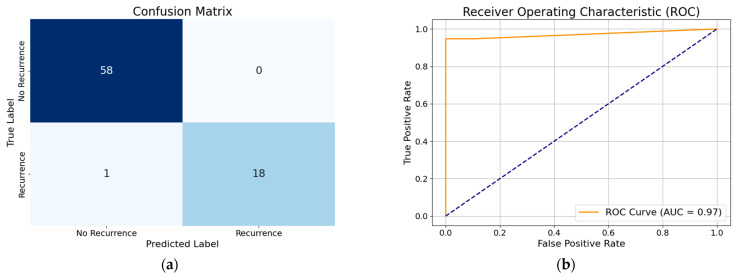
(**a**) Confusion matrix of optimized XGBoost model by basic whale optimization algorithm with all features; (**b**) ROC curve of optimized XGBoost model by basic whale optimization algorithm with all features.

**Figure 6:**
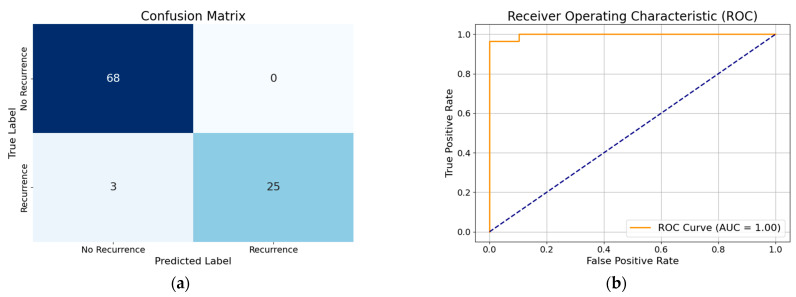
(**a**) Confusion matrix of optimized XGBoost model by modified whale optimization algorithm with all features; (**b**) ROC curve of optimized XGBoost model by modified whale optimization algorithm with all features.

**Figure 7:**
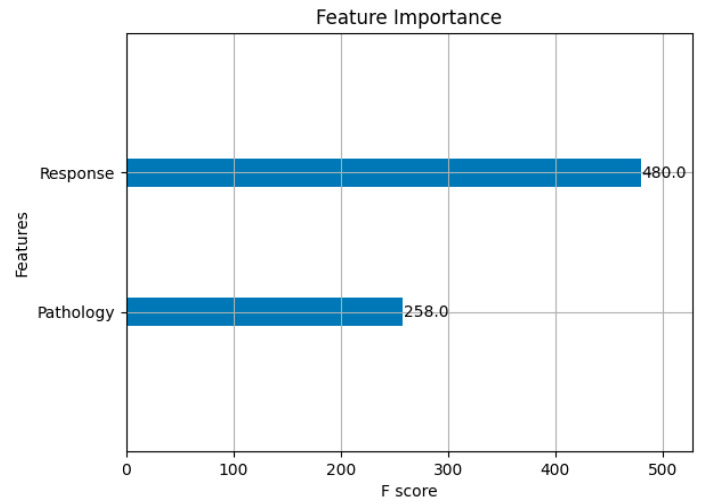
Importance of selected features.

**Figure 8:**
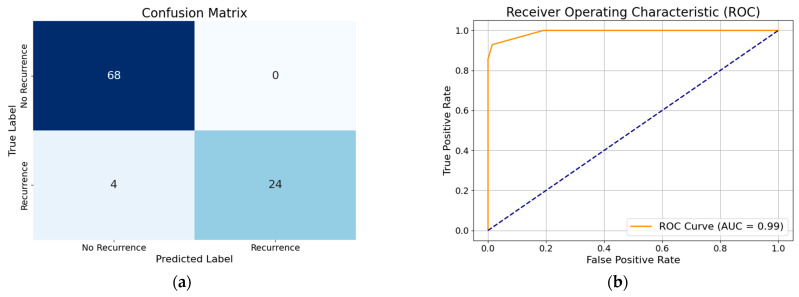
(**a**) Confusion matrix of optimized XGBoost model by modified whale optimization algorithm with selected features; (**b**) ROC curve of optimized XGBoost model by modified whale optimization algorithm with selected features.

**Figure 9:**
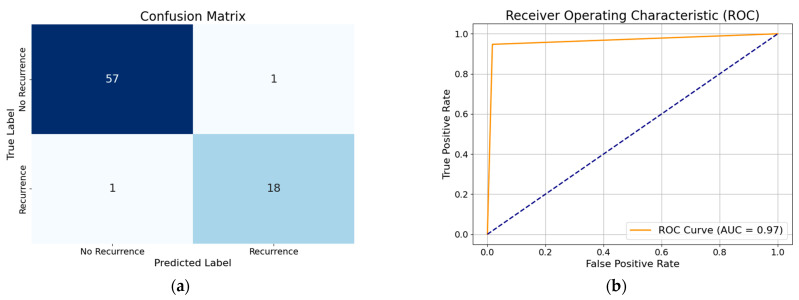
(**a**) Confusion matrix of optimized XGBoost model by basic whale optimization algorithm with selected features; (**b**) ROC curve of optimized XGBoost model by basic whale optimization algorithm with selected features.

The performance of the XGBoost model with all features demonstrates high accuracy (96%), indicating that the model correctly classifies the majority of cases. The precision (94%) suggests that when the model predicts recurrence, it is correct 94% of the time, minimizing false positives. The recall (96%) shows that the model successfully identifies 96% of actual recurrence cases, reducing false negatives. The F1-score (95%), which balances precision and recall, confirms the model’s overall effectiveness in predicting DTC recurrence. By analyzing the performance of the WOA-based optimized XGBoost classifier without any feature reduction, we gain an accuracy of about 99%. The XGBoost model with all features, when optimized using hyperparameter tuning with the modified WOA, showed improved accuracy (97%). This indicates enhanced classification performance. The precision (100%) suggests that all positive predictions were correct, eliminating false positives. The recall (89%) is slightly lower compared to the precision (100%), which suggests that while the model is highly confident in its positive predictions, i.e., it rarely misclassifies non-recurrence cases as recurrence, it is missing some recurrent cases. The F1-score (94%) reflects a balanced performance, maintaining overall model efficiency. Visual representation of each model’s classification performance are provided via a confusion matrix and ROC curve (Figure 4, Figure 5, Figure 6, Figure 7, Figure 8 and Figure 9).

### 3.2. Performance of XGBoost Model with Selected Features

In this case we employ Binary Encoding for feature selection. In that context, solutions (or whales) are represented as binary vectors. Every value inside the vector corresponds to the feature; 1 means that the feature is included, and 0 means that the feature is missed. For example, if there are 10 features while voting a whale that look like [1, 0, 1, 1, 0, 0, 1, 0, 1, 0] then the whale selects features 1, 3, 4, 7, and 9. Initially, the algorithm sets generated population of whales (solutions) using the chaotic map that will not overlap on each other in terms of initial selected features. In the subsequent feature selection phase, during the voting process, a whale represented as a binary vector such as [1, 0, 1, 1, 0, 0, 1, 0, 1, 0] indicates the selection of features corresponding to the positions with a value of 1. In this example, the whale selects features 1, 3, 4, 7, and 9. Initially the algorithm sets generated populations of whales (solutions) using the chaotic map that will not overlap on each other in terms of initial selected features. In subsequent iterations, the solutions are modified using the encircling prey and bubble-net attacking modes. The fitness function also ranks each solution according to the F1-score of the XGBoost classifier, and the best solution is updated iteratively. The algorithm continues to run either until it reaches a predefined number of iterations or until no further improvements are observed in the solution. The most optimal whale at the end of the process represents the best-performing configuration of features, constituting the final selected feature subset. In our context the selected feature includes “Pathology” and “Response”. This is represented in Figure 7. Our MWOA selected only two key features (‘Pathology’ and ‘Response’). This significantly reduced model complexity and the risk of overfitting.

“Response” has the highest importance score (480), followed by “Pathology” (258), indicating their significant contribution to the model’s performance. The feature “Response” refers to how a patient initially responds to treatment. Patients with an incomplete response are at a much higher risk of recurrence. Hence, it is one the most influential factors in DTC prediction and classification. Likewise, the feature “Pathology” denotes the specific type and behavior of the tumor. Certain aggressive tumor types and factors like tumor size, spread to lymph nodes, and invasion into nearby tissues increase the chances of recurrence. For this reason, Pathology is also an important feature for DTC recurrence prediction and classification.

By analyzing the performance of the MWOA-based, optimized XGBoost model with selected features, we gain an accuracy of about 96%. Similarly, from the performance of the WOA-based XGBoost model with selected features we obtain an accuracy of 96%, precision of 97%, recall of 93%, and F1 score of 95%. To further visualize the classification performance, the confusion matrix and ROC curve of the optimized XGBoost model by way of the modified WOA with selected features and the optimized XGBoost model by way of the basic WOA with selected features are shown in Figure 8 and Figure 9.

### 3.3. Overfitting Prevention and Model Robustness Evaluation

Overfitting and model generalization have been carefully addressed in our study through several strategies:

- Feature Selection: Our Modified WOA selected only two key features (‘Pathology’ and ‘Response’). This significantly reduced model complexity and the risk of overfitting.
- Hyperparameter Optimization: The WOA also tuned important XGBoost parameters such as max_depth and learning_rate, which are known to control model complexity and regularization.
- Fitness Function with Regularization: Our fitness function included a penalty for using more features, encouraging the model to favor simpler, more generalizable solutions.
- Cross-Validation: We used 5-fold cross-validation during training to evaluate the performance of each candidate model configuration. This provided a robust estimate of generalization ability. The best configuration achieved a mean F1-score of 0.8823 ± 0.0505 on the training data.
- Independent Test Set Evaluation: After optimization, the final model was tested on an unseen dataset, achieving an F1-score of 0.9231 and accuracy of 0.9583, confirming strong generalization to new data.

To assess the statistical significance of the achieved results, we have also employed 5-fold cross-validation during the optimization process. This approach provides a distribution of performance scores (e.g., F1-score), allowing us to report both the mean and standard deviation (Mean F1-score = 0.8823 ± 0.0505). The relatively low variance indicates consistent performance across different data partitions, supporting the reliability of the results. Additionally, the final model was evaluated on an independent test set, yielding an F1-score of 0.9231 and accuracy of 0.96. The consistency between cross-validation results and independent test performance suggests that the model’s effectiveness is not due to random chance or overfitting but reflects statistically reliable performance.

### 3.4. Model Performance Comparison

From Table 2 we can observe that the baseline model without hyperparameter tuning performs decently, but there is room for improvement. So hyperparameter tuning was performed using both the basic WOA and its modified version. Hyperparameter tuning using the basic WOA significantly improves all metrics, especially accuracy and F1-score. The modified WOA improves precision to 100%, but recall drops to 89%, suggesting a trade-off where the model is highly confident in its predictions but may miss some positive cases. Feature selection slightly reduces accuracy but maintains 100% precision, though recall further drops to 85%, indicating that the model may be too conservative in identifying positive cases.

**Table 2:** Model performance comparison.

Model	Accuracy	Precision	Recall	F1 Score	AUC-ROC
XGBoost with all features	96%	94%	96%	95%	1.00
Optimized XGBoost by basic WOA with all features	99%	99%	97%	98%	0.97
Optimized XGBoost by modified WOA with all features	97%	100%	89%	94%	1.00
Optimized XGBoost by modified WOA with selected features	96%	100%	85%	92%	0.99
Optimized XGBoost by basic WOA with selected features	96%	97%	93%	95%	0.99

Therefore, we can ultimately conclude that the XGBoost model hyperparameter tuned with the basic WOA with all features achieves the highest accuracy (99%) and a strong balance across precision, recall, and F1-score. The models hyperparameter-tunned with the modified WOA (both with all and selected features) achieve 100% precision, meaning no false positives. However, their lower recall indicates a higher rate of false negatives. Reducing the feature set (while using the MWOA) lowers recall further, showing that certain features are crucial for detecting all positive cases. Though it has lower recall, its high AUC-ROC score indicates outstanding overall discriminative ability. This suggests that, although a small proportion of recurrence cases were not identified (reflecting the trade-off in recall), the model was still highly capable of distinguishing between recurrence and non-recurrence cases across thresholds. Another reason for lower recall might also be due to the addition of the weight inertia and PWLCW mechanisms in the MWOA which are designed to improve convergence stability and avoid local optima. However, these enhancements may also make the search process more conservative, potentially affecting the model’s ability to identify a broader range of true positives, especially borderline cases. While the MWOA improves global optimization by better balancing exploration and exploitation, it may under-explore specific regions of the feature space associated with rare positive cases, which are critical for achieving higher recall. The MWOA-optimized XGBoost model achieved perfect precision (100%) but a slightly lower recall (89%). This means that while all predicted recurrence cases were correct, around 11% of actual recurrence cases were missed, translating to approximately 13 patients out of 120 recurrence cases. In the context of DTC, where every missed recurrence is critical, this is an important consideration.

However, achieving a perfect balance between precision and recall is challenging, especially in clinical applications. A model with very high recall may flag more patients incorrectly, increasing unnecessary follow-ups and anxiety. Our model, by contrast, makes highly confident predictions with zero false positives. This trade-off reflects a deliberate focus on reliability and trustworthiness in positive predictions. While missing some recurrence cases is not ideal, a recall of 89% is still within an acceptable range for many clinical prediction models particularly when supported by clinical oversight. This model is intended to aid, not replace, physician judgment and can be effectively used to flag high-risk patients with strong confidence.

In addition to predictive performance, computational efficiency is another key consideration when selecting optimization techniques for real-world applications. Notably, the MWOA demonstrated a significant advantage in terms of optimization time. When applied to the model using all features, the total optimization time for the MWOA was only 0.15 s, compared to 16.66 s for the standard WOA. This drastic reduction highlights the MWOA’s ability to reach high-quality solutions more quickly, making it more suitable for clinical environments where rapid model tuning or retraining may be required. The improved convergence speed of the MWOA which was achieved through enhanced search strategies like inertia weighting and chaotic initialization further adds value to its practical deployment.

### 3.5. Comparison with Previous Works

Table 3 compares the performance of our proposed models with other existing models such as RF and MLR used by researchers on the same dataset in DTC recurrence prediction. Our proposed model outperforms all other models. The XGBoost model optimized with the WOA achieved the highest accuracy (99%) with all features (All f.), surpassing other models like RF [10,11] and MLR [9]. The F1-score (98%) indicates a balanced performance, ensuring a strong trade-off between precision and recall. Precision (99%) indicates that the model does not falsely classify any non-recurrence cases as recurrence, which is critical for reducing unnecessary interventions.

RF performs well but is slightly less effective than our optimized XGBoost model. MLR is significantly weaker, confirming that non-linear models are better suited for this problem.

We have also compared our proposed method with recent studies on thyroid disorder classification. While the results reported in [15,16] may appear to outperform ours based on certain metrics, there are several key differences to consider. First, our study specifically targets the prediction of recurrence in DTC which is a more clinically complex task than general thyroid disorder classification. This problem involves detecting subtle patterns in the data and dealing with the relatively low prevalence of recurrence. This makes it inherently more challenging to achieve high accuracy. Second, we introduced a customized WOA to fine-tune model hyperparameters. This approach strikes a balance between exploration and exploitation that leads to a well-regularized model that resists overfitting and performs robustly on unseen data.

These results highlight the potential of the WOA-optimized XGBoost model as an effective tool for predicting DTC recurrence. It has the potential to be a practical model for real-world clinical applications, providing valuable insights to enhance patient outcomes.

While previous studies such as Borozooei et al. [8] and Latif et al. [13] have explored machine learning models for thyroid disease classification and recurrence prediction, our work offers a distinct contribution through the integration of a XGBoost model optimized using both the standard WOA and its enhanced variant, the MWOA. The modifications introduced in the MWOA include inertia weight and piecewise linear chaotic maps, which are designed to improve the optimizer’s ability to balance exploration and exploitation. This not only enhances the precision of hyperparameter tuning and feature selection but also helps the model avoid common issues such as premature convergence. From a clinical perspective, this improved optimization translates to more reliable predictions, particularly in identifying high-risk patients with minimal false positives. On the computational side, the MWOA demonstrates superior performance compared to traditional algorithms like particle swarm optimization (PSO) and the genetic algorithm (GA) by maintaining solution diversity and achieving better convergence. Unlike PSO, which is known for fast convergence but often struggles with local optima in complex datasets, the MWOA maintains diversity and stability throughout the search process. Similarly, although the GA provides strong search capabilities via crossover and mutation, it can be slower and more computationally demanding. In contrast, the MWOA’s biologically inspired mechanisms such as encircling prey and bubble-net attacking, allow for dynamic control between exploration and exploitation leading to faster convergence.

This is particularly valuable in medical domains like DTC recurrence prediction, where subtle and complex patterns must be captured with precision. Notably, our results show that the MWOA reduced optimization time to just 0.15 s when using all features, indicating its potential suitability for real-time clinical applications. These combined strengths make the MWOA not only efficient for hyperparameter optimization and feature selection but also highly robust in building generalizable models for complex biomedical problems. The model’s strong performance, achieving an accuracy of 96% and 97%, further reinforces its promise for practical deployment in healthcare environments.

## 4. Conclusions and Future Work

Differentiated thyroid cancer (DTC) is the most common type of thyroid cancer and it does not always behave the same for all patients. While some recover completely after treatment, others may experience a recurrence even after undergoing surgery and radioactive iodine therapy. This recurrence is a concern and can complicate treatment strategies and affect long-term patient survival. So, predicting it accurately is vital for the patient. The objectives of our research were (i) to investigate the DTC dataset and extract important features required for predicting the recurrence of DTC; (ii) to optimize the hyperparameters of the Extreme Gradient Boosting (XGBoost) model using both a modified whale optimization algorithm (WOA) and the basic WOA; (iii) to investigate the performance of the optimized XGBoost model for predicting the recurrence of DTC; and (iv) to make a comparison between the outcome of our study and prior research. In order to accomplish this goal, we have investigated the UCI dataset and extracted some important features for DTC recurrence prediction. To improve the prediction performance, we have tuned the hyperparameters of the XGBoost model using both a modified and the basic WOA. For further validation, we have compared our optimized model with prior works. Our model, when trained with all features, has shown an accuracy of 99% when its hyperparameter was tuned using the basic WOA and 97% when its hyperparameter was tuned using a modified WOA. The basic-WOA-based, optimized XGBoost model performs better than the modified-WOA-optimized version. This could be because the dataset was not well suited for the modifications introduced in the WOA algorithm. However, our model performed exceptionally well compared to other models trained using the same DTC dataset. Our model has exhibited impressive accuracy in predicting DTC recurrence. From a healthcare perspective, we hope this study contributes significantly to DTC recurrence prediction. With the help of our model, DTC recurrence can be predicted efficiently and also in a cost-effective manner. The high accuracy of our model (up to 99%) suggests strong potential for integration into thyroid cancer surveillance protocols. In clinical practice, such a model could assist in early identification of patients at higher risk of recurrence, allowing for more personalized monitoring and timely intervention. For example, patients flagged as high risk by the model could undergo more frequent follow-ups, imaging, or lab evaluations, while those at lower risk might avoid unnecessary procedures. This helps to optimize both patient outcomes and healthcare resources. Given the model’s rapid processing time (0.15 s), it could be incorporated into electronic health record (EHR) systems or cloud-based diagnostic platforms to support real-time clinical decision-making. While additional validation in diverse patient populations is necessary, this model offers a promising step toward more proactive and cost-effective DTC management.

While this research successfully lays a foundation for a robust model for DTC recurrence prediction, certain limitations present opportunities for future research. One of the key limitations is that the model was developed using data from a single-center cohort, which may not fully reflect the diversity of real-world clinical populations. The relatively small and homogenous dataset may also affect the model’s ability to generalize more broadly. To address this, future research should focus on testing the model with larger, more varied datasets collected from multiple hospitals or institutions. Doing so would help evaluate how well the model performs across different settings and patient groups. It is also important to consider how this kind of predictive tool can be integrated into everyday clinical practice, for instance, by embedding it in systems that help doctors identify patients at higher risk of recurrence. This step is crucial to making the model both clinically relevant and practically useful.

Moreover, though the basic WOA demonstrated superior performance, some advancement in optimization techniques could further refine the predictive accuracy. Investigating advanced metaheuristic algorithms such as Hybrid WOA, GA [32], PSO [33], and Differential Evolution (DE) [34] could provide better feature selection and hyperparameter tuning strategies. Another crucial extension can be implementing this model into a real-time monitoring system for DTC recurrence prediction that could allow for continuous assessment of patient risk based on longitudinal, clinical and imaging data. This integration could be achieved using cloud-based AI systems and Internet of Medical Things (IoMT) frameworks, enabling healthcare providers to receive automated alerts for high-risk patients [35].

Lastly, future studies could address the explainability of AI models in clinical applications. Techniques such as SHapley Additive exPlanations (SHAP) [36] and Local Interpretable Model-Agnostic Explanations (LIMEs) [37] could be combined with the MWOA which can enhance model transparency, ensuring clinicians understand the decision-making process behind AI-driven predictions [36]. These methods can offer both broad and case-specific explanations, helping clinicians better understand how different features contribute to the model’s predictions. Including such techniques in future versions of the model could support greater transparency and build the trust needed for clinical adoption, particularly in sensitive areas like cancer recurrence prediction. By addressing these limitations and incorporating real-time monitoring, the proposed systems could revolutionize DTC recurrence prediction.

## Figures and Tables

**Table 1:** Summary of patient data for differentiated thyroid cancer recurrence.

Category	Details	Count
Total samples	-	383
Recurrence	-	120
Cancer Stages	Stage I	333
	Stage II	32
	Stage III	4
	Stage IV A	3
	Stage IV B	11
Pathology	Papillary Carcinoma	287
	Micro Papillary Carcinoma	48
	Follicular Carcinoma	28
	Hurthel Cell Carcinoma	20
Tumor Size (T)	T0	0
	T1	92
	-T1a	49
	-T1b	43
	T2	149
	T3	110
	-T3a	94
	-T3b	16
	T4	26
Lymph Node (N)	N0	264
	N1	119
	N2	0
	N3	0
Metastasis (M)	M0	365
	M1	18

**Table 3:** Comparison with previous works.

Work	Dataset Used	Model Used	Accuracy	F1-Score	Precision	Recall
[10]	DTC (All f.) [8]	RF	96%	94%	94%	94%
[9]	DTC (All f.) [8]	MLR	78.32%	-	-	-
[11]	DTC (All f.) [8]	RF	95%	95%	95%	96%
[15]	KEEL and hypothyroid dataset	SL ensemble	99.602%	98.565%	99.785%	97.413%
[16]	DHQ [31]	SFM	100%	100%	100%	100%
Our	DTC (All f.) [8]	XGBoost	99%	98%	99%	97%

All f.—All Features; SFM—Select From Model.

## Data Availability

The raw data supporting the conclusions of this research will be made available by the authors upon request.

## Abbreviations

The following abbreviations are used in this manuscript:

DTC	Differentiated thyroid cancer
WOA	Whale optimization algorithm
AI	Artificial intelligence
PWLCM	Piecewise linear chaotic map

## References

[1] Mazzaferri E.L. (2005). Empirically treating high serum thyroglobulin levels. J. Nucl. Med..

[2] Burns W.R., Zeiger M.A. (2010). Differentiated thyroid cancer. Seminars in Oncology.

[3] Schlumberger M., Leboulleux S. (2021). Current practice in patients with differentiated thyroid cancer. Nat. Rev. Endocrinol..

[4] Haugen B.R. (2017). 2015 American Thyroid Association management guidelines for adult patients with thyroid nodules and differentiated thyroid cancer: What is new and what has changed?. Cancer.

[5] Hollenbeak C.S., Boltz M.M., Schaefer E.W., Saunders B.D., Goldenberg D. (2013). Recurrence of differentiated thyroid cancer in the elderly. Eur. J. Endocrinol..

[6] Zahedi A., Bondaz L., Rajaraman M., Leslie W.D., Jefford C., Young J.E., Pathak K.A., Bureau Y., Rachinsky I., Badreddine M. (2020). Risk for thyroid cancer recurrence is higher in men than in women independent of disease stage at presentation. Thyroid.

[7] Medas F., Canu G.L., Boi F., Lai M.L., Erdas E., Calò P.G. (2019). Predictive factors of recurrence in patients with differentiated thyroid carcinoma: A retrospective analysis on 579 patients. Cancers.

[8] Borzooei S., Briganti G., Golparian M., Lechien J.R., Tarokhian A. (2024). Machine learning for risk stratification of thyroid cancer patients: A 15-year cohort study. Eur. Arch. Oto-Rhino-Laryngol..

[9] Chattopadhyay S. (2024). Towards Predicting Recurrence Risk of Differentiated Thyroid Cancer with a Hybrid Machine Learning Model. Medinformatics.

[10] Setiawan K.E. (2024). Predicting recurrence in differentiated thyroid cancer: A comparative analysis of various machine learning models including ensemble methods with chi-squared feature selection. Commun. Math. Biol. Neurosci..

[11] Clark E., Price S., Lucena T., Haberlein B., Wahbeh A., Seetan R. (2024). Predictive Analytics for Thyroid Cancer Recurrence: A Machine Learning Approach. Knowledge.

[12] Rajni G., Pankaj R. (2024). XGBoost for Heart Disease Prediction: Achieving High Accuracy with Robust Machine Learning Techniques. Int. J. Innov. Sci. Eng. Manag..

[13] Latif M.A., Mushtaq Z., Arif S., Rehman S., Qureshi M.F., Samee N.A., Alabdulhafith M., Al-masni M.A. (2024). Improving Thyroid Disorder Diagnosis via Ensemble Stacking and Bidirectional Feature Selection. Comput. Mater. Contin..

[14] Oture O., Iqbal M.Z., Wang X. (2025). Enhanced diagnosis of thyroid diseases through advanced machine learning methodologies. Sci.

[15] Afshan N., Mushtaq Z., Alamri F.S., Qureshi M.F., Khan N.A., Siddique I. (2023). Efficient thyroid disorder identification with weighted voting ensemble of super learners by using adaptive synthetic sampling technique. AIMS Math..

[16] Akhtar T., Gilani S.O., Mushtaq Z., Arif S., Jamil M., Ayaz Y., Butt S.I., Waris A. (2021). Effective voting ensemble of homogenous ensembling with multiple attribute-selection approaches for improved identification of thyroid disorder. Electronics.

[17] Mirjalili S., Lewis A. (2016). The whale optimization algorithm. Adv. Eng. Softw..

[18] García S., Luengo J., Herrera F. (2015). Data Preprocessing in Data Mining.

[19] Hastie T. (2009). The elements of statistical learning: Data mining, inference, and prediction. J. Am. Stat. Assoc..

[20] Kuhn M. (2013). Applied Predictive Modeling.

[21] Chakraborty S., Saha A.K., Chakraborty R., Saha M. (2021). An enhanced whale optimization algorithm for large scale optimization problems. Know.-Based Syst..

[22] Shi Y., Eberhart R. A modified particle swarm optimizer. Proceedings of the 1998 IEEE International Conference on Evolutionary Computation Proceedings. IEEE World Congress on Computational Intelligence (Cat. No.98TH8360).

[23] Guo W., Liu T., Dai F., Xu P. (2020). An Improved Whale Optimization Algorithm for Feature Selection. Comput. Mater. Contin..

[24] Chao I.M., Hsiung S.-C., Liu J.-L. (2020). Improved Whale Optimization Algorithm Based on Inertia Weights for Solving Global Optimization Problems. Adv. Technol. Innov..

[25] Saiki Y., Takahasi H., Yorke J.A. (2021). Piecewise linear maps with heterogeneous chaos. Nonlinearity.

[26] Alzahrani J.S., Rizwanullah M., Osman A.E. (2023). Piece-Wise Linear Chaotic Mapping-based Beluga Whale Optimization Algorithm-based Indoor Activity Monitoring for Elderly and Visually Impaired Persons. J. Disabil. Res..

[27] Friedman J.H. (2001). Greedy function approximation: A gradient boosting machine. Ann. Stat..

[28] Chen T., Guestrin C. Xgboost: A scalable tree boosting system. Proceedings of the 22nd Acm Sigkdd International Conference on Knowledge Discovery and Data Mining.

[29] Nandini S. (2024). Comparative Study of Machine Learning Algorithms in Detecting Cardiovascular Diseases. arXiv.

[30] Ke G., Meng Q., Finley T., Wang T., Chen W., Ma W., Ye Q., Liu T.-Y. (2017). Lightgbm: A highly efficient gradient boosting decision tree. Adv. Neural Inf. Process. Syst..

[31] Abbad Ur Rehman H., Lin C.-Y., Mushtaq Z. (2021). Effective K-nearest neighbor algorithms performance analysis of thyroid disease. J. Chin. Inst. Eng..

[32] Mirjalili S., Mirjalili S. (2019). Genetic algorithm. Evolutionary Algorithms and Neural Networks. Studies in Computational Intelligence.

[33] Kennedy J., Eberhart R. Particle swarm optimization. Proceedings of the ICNN’95—International Conference on Neural Networks.

[34] Feoktistov V. (2006). Differential Evolution.

[35] Hossain M.S., Muhammad G., Guizani N. (2020). Explainable AI and mass surveillance system-based healthcare framework to combat COVID-I9 like pandemics. IEEE Netw..

[36] Lundberg S. (2017). A unified approach to interpreting model predictions. arXiv.

[37] Ribeiro M.T., Singh S., Guestrin C. Anchors: High-precision model-agnostic explanations. Proceedings of the AAAI Conference on Artificial Intelligence.

